# Characterization of Transferrable Mechanisms of Quinolone Resistance (TMQR) among Quinolone-resistant *Escherichia coli* and *Klebsiella pneumoniae* causing Urinary Tract Infection in Nepalese Children

**DOI:** 10.1186/s12887-023-04279-5

**Published:** 2023-09-13

**Authors:** Raj Kumar Shrestha, Ashmita Thapa, Dhruba Shrestha, Sabi Pokhrel, Anubhav Aryal, Rupika Adhikari, Nipun Shrestha, Bhim Gopal Dhoubhadel, Christopher M Parry

**Affiliations:** 1 Siddhi Memorial Hospital, Bhaktapur, Nepal; 2 School of Tropical Medicine and Global Health (TMGH), Nagasaki University, Nagasaki, Japan; 3 Department of Respiratory Infections, Institute of Tropical Medicine, Nagasaki University, Nagasaki, Japan; 4 Clinical Sciences, Liverpool School of Tropical Medicine, Liverpool, UK; 5 Centre for Tropical Medicine and Global Health, Nuffield Department of Medicine, University of Oxford, Oxford, UK

**Keywords:** Transferrable mechanisms of quinolone resistance (TMQR), Quinolone resistance, Urinary tract infection, Children, Nepal

## Abstract

**Background:**

Transferrable mechanisms of quinolone resistance (TMQR) can lead to fluoroquinolone non-susceptibility in addition to chromosomal mechanisms. Some evidence suggests that fluoroquinolone resistance is increasing among the pediatric population. We sought to determine the occurrence of TMQR genes among quinolone-resistant *E. coli* and *K. pneumoniae* causing urinary tract infections among Nepalese outpatient children (< 18 years) and identify molecular characteristics of TMQR-harboring isolates.

**Methods:**

We performed antimicrobial susceptibility testing, phenotypic extended-spectrum β-lactamase (ESBL) and modified carbapenem inactivation method tests, and investigated the presence of six TMQR genes (*qnrA*, *qnrB*, *qnrS*, *aac(6’)-Ib-cr*, *oqxAB, qepA*), three ESBL genes (*bla*_CTX−M_, *bla*_TEM_, *bla*_SHV_), and five carbapenemase genes (*bla*_NDM_, *bla*_OXA−48_, *bla*_KPC_, *bla*_IMP_, *bla*_VIM_). The quinolone resistance-determining region (QRDR) of *gyrA* and *parC* were sequenced for 35 TMQR-positive isolates.

**Results:**

A total of 74/147 (50.3%) isolates were TMQR positive by multiplex PCR [*aac(6’)-Ib-cr* in 48 (32.7%), *qnrB* in 23 (15.7%), *qnrS* in 18 (12.3%), *qnrA* in 1 (0.7%), and *oqxAB* in 1 (0.7%) isolate]. The median ciprofloxacin minimum inhibitory concentration of TMQR-positive isolates (64 µg/mL) was two-fold higher than those without TMQR (32 µg/mL) (p = 0.004). Ser-83→Leu and Asp-87→Asn in GyrA and Ser-80→Ile in ParC were the most common QRDR mutations (23 of 35). In addition, there was a statistically significant association between TMQR and two β-lactamase genes; *bla*_CTX−M_ (p = 0.037) and *bla*_TEM_ (p = 0.000).

**Conclusion:**

This study suggests a high prevalence of TMQR among the quinolone-resistant *E. coli* and *K. pneumoniae* isolates causing urinary tract infection in children in this area of Nepal and an association with the carriage of ESBL gene. This is a challenge for the management of urinary infections in children. Comprehensive prospective surveillance of antimicrobial resistance in these common pathogens will be necessary to devise strategies to mitigate the emergence of further resistance.

**Supplementary Information:**

The online version contains supplementary material available at 10.1186/s12887-023-04279-5.

## Background

Fluoroquinolone (FQ) antimicrobials are important in the treatment of a range of infections, including urinary tract infections (UTIs). They have a broad spectrum of activity, high bioavailability, convenient dosing regimens, and high potency [1, 2]. FQs are listed as an essential medicine by World Health Organization (WHO) [3]. Their extensive use has led to a marked increase in FQ resistance globally [4–7].

Quinolone/fluoroquinolone (Q/FQ) resistance in *Enterobacterales* is commonly attributed to chromosomal mutations in the quinolone resistance-determining region (QRDR) of the genes encoding subunits of DNA gyrase (GyrA and GyrB) and topoisomerase IV (ParC and ParE) [8]. The reduction of Q/FQ concentration in the cytoplasm by chromosomal efflux pumps or permeability alterations also contributes to resistance [9]. Transferrable mechanisms of quinolone resistance (TMQR) can additionally confer low-level Q/FQ resistance and promote the development of full resistance [10]. TMQR determinants include seven Qnr proteins, AAC(6’)-Ib-cr (an aminoglycoside acetyltransferase), and two efflux pumps, QepA and OqxAB. Qnr proteins (QnrA, QnrB, QnrC, QnrD, QnrE, QnrS, and QnrVC) are dimeric proteins belonging to the pentapeptide repeat protein (PRP) family and protect DNA gyrase and topoisomerase IV from the action of quinolones [11]. The AAC(6’)-Ib-cr is a bifunctional variant of AAC(6’)-Ib that imparts resistance to aminoglycosides and fluoroquinolones having a piperazinyl substituent, such as ciprofloxacin and norfloxacin, via acetylation of amino nitrogen in the piperazinyl ring [11]. These diverse mechanisms can act in concert to confer non-susceptibility to Q/FQ.

As the use of FQ is restricted in children [12], the increase in FQ resistance among the pediatric population is important [13, 14]. In a recent study from our institution, 66 (41.8%) of 158 *E. coli* and 7 (23.3%) of 30 *K. pneumoniae* isolates causing UTI among children were resistant to ofloxacin [15]. Studies from tertiary care centers of Nepal focusing on pediatric UTI have reported ciprofloxacin resistance in 576 (78%) of 739 and 44 (63%) of 69 isolates, and ofloxacin resistance in 104 (62%) of 168 *E. coli* isolates [16–18]. We have investigated the occurrence of TMQR among quinolone-resistant *E. coli* and *K. pneumoniae* isolates causing UTI among children at our institution and sought to identify molecular characteristics of TMQR-harboring isolates.

## Materials and methods

### Study design and setting

This is a retrospective study conducted at Siddhi Memorial Hospital (SMH), Bhaktapur, Nepal. SMH is a 50-bedded secondary care maternal and pediatric hospital with 10 pediatric ICU beds, serving about 16,000 pediatric OPD visits annually. *E. coli* and *K. pneumoniae* isolates obtained from UTI patients less than 18 years old attending the outpatient department (OPD) of the hospital were included in the study. An anonymized dataset, with personal identifiers removed, containing the patient’s age, sex, name of the pathogen, and the susceptibility result to nalidixic acid from June 2018 to February 2021 was retrieved from the microbiology laboratory.

### Microbiological methods at the time of isolation of the isolates

Clean catch mid-stream urines were collected from children suspected of UTI as per the pediatrician’s discretion as a part of routine patient diagnosis. Urine cultures were performed by semi-quantitative method on a cysteine-lactose-electrolyte deficient agar (CLED) plates which were then incubated at 37 ^o^C for 18–24 h aerobically. Urine cultures with a growth of ≥ 10^5^ CFU/mL were considered for further processing.

The presumptive identification of the pathogens was performed by Gram stain, colony morphology, and a panel of in-house biochemical tests. Susceptibility to nalidixic acid (NA) was performed by the Kirby Bauer disk diffusion method [19]. Significant isolates were stored at -40 ^o^C at the time of isolation.

*E. coli* and *K. pneumoniae* isolate resistant to NA were sub-cultured from the frozen stocks on a MacConkey agar and sheep blood agar till uniform well-isolated colonies were obtained. The investigations carried out in this study include antimicrobial susceptibility testing and molecular investigations for the detection of β-lactamases, TMQR genes, and mutations in *gyrA* and *parC*.

### Antimicrobial susceptibility testing

Antimicrobial susceptibility testing (AST) of *E. coli* and *K. pneumoniae* isolates was performed by the Kirby Bauer disk diffusion method according to the CLSI guideline [19]. The antimicrobial disks used for testing were ampicillin (10 µg) [tested only for *E. coli*], amoxicillin-clavulanic acid (20/10 µg), piperacillin-tazobactam (100/10 µg), cefazolin (30 µg), cefuroxime (30 µg). cefixime (5 µg), cefotaxime (30 µg), ceftazidime (30 µg), cefepime (30 µg), imipenem (10 µg), ciprofloxacin (5 µg), trimethoprim sulphamethoxazole (1.25/23.75 µg), nitrofurantoin (300 µg), and amikacin (30 µg). Amoxicillin-clavulanic acid, piperacillin-tazobactam, ceftazidime, ciprofloxacin, and imipenem disks were purchased from the manufacturer Mast (Mast group Ltd, Liverpool, UK) with the remainder from HiMedia (HiMedia, India). The minimum inhibitory concentration (MIC) of ciprofloxacin was determined by E-test (0.002-32 µg/mL) (HiMedia, India), and those isolates with MIC ≥ 32 µg/mL were further tested by agar dilution method following the procedures described by CLSI [20]. An isolate was defined to display multidrug resistance (MDR) if non-susceptible to ≥ 1 agent in ≥ 3 antimicrobial categories [21]. *Escherichia coli* ATCC 25922 was used for quality control.

### Nucleic acid extraction

The genomic DNA was extracted using Qiagen DNA mini kit (Qiagen, Hilden, Germany) following the procedures described by the manufacturer with the only exception that the final elution was made with 150 µl of nuclease-free water (NFW). The DNA extracts were quantified using Qubit 4 Fluorometer (Invitrogen, Thermo Fisher Scientific) following the manufacturer’s recommendations.

### Characterization of β-lactamases

The phenotypic determination of extended-spectrum β-lactamase (ESBL) production was first performed by a combination disc diffusion method with cefotaxime, cefotaxime-clavulanic acid, ceftazidime, and ceftazidime-clavulanic acid (D62C and D64C, Mast group Ltd, Liverpool, UK). The results were interpreted as described in the CLSI guideline [19]. DNA samples of the ESBL-positive isolates were analyzed by PCR to detect *bla*_CTX−M_ [22], *bla*_SHV_ (for *E. coli* only), and *bla*_TEM_ by the assays described elsewhere [23].

The modified carbapenem inactivation method (mCIM) was used to confirm carbapenemase production among imipenem non-susceptible isolates as described in the CLSI guideline [19]. DNA samples of these isolates were analyzed by PCR to detect *bla*_NDM_, *bla*_OXA−48_, *bla*_KPC_, *bla*_IMP_, and *bla*_VIM_ using the primers published previously [24].

*Escherichia coli* ATCC 25922 and clinical strains confirmed to harbor *bla*_CTX−M_, *bla*_TEM_, and *bla*_SHV_ β-lactamase genes were used for quality control for ESBL phenotyping and genotyping. Previously characterized strains confirmed to harbor *bla*_NDM_ and *bla*_OXA−48_ were used as positive controls for mCIM. DNA extracted from the control strains was used as a positive control, and *Escherichia coli* ATCC 25922 DNA was used as a negative control in PCR assays.

### Detection of TMQR genes

Previously validated multiplex PCR assay for the detection of TMQR genes was used for the detection of *qnrA, qnrB, qnrS, oqxAB* (reported only for *E. coli*), *qepA*, and *aac(6’)-Ib-cr* [25]. Briefly, the PCR reaction mixture of 50 µl was prepared with 25 µl of multiplex PCR master mix (2X) (Qiagen, Hilden, Germany), 5 µl of the pool of primers containing 2 µM of each primer, 5 µl of template of concentration of 20 ng/µl, and 15 µl of NFW (Ambion™ Nuclease-Free water, Invitrogen, Thermo Fisher Scientific). The PCR amplification was carried out in Veriti 96 Well Thermal Cycler (appliedbiosystems, Thermo Fisher Scientific) with 15 min of initial denaturation at 95 ^o^C followed by 30 cycles of denaturation at 94 ^o^C for 30 s, annealing at 63 ^o^C for 90 s, and extension for 10 min at 72 ^o^C. The amplification products were first resolved by gel electrophoresis (1.5%, w/v) at 100 V for 40 min and visualized in a gel documentation system (Major Science, California, USA). All PCR amplicons of *qnr* genes were sequenced and confirmed by BLAST (Basic Local Alignment Search Tool).

### **Detection of mutations in *gyrA *and *parC***

A convenience sample of thirty-five TMQR-positive isolates with representative ciprofloxacin’s interpretive categories (susceptible, intermediate, and resistant) for *E. coli* and *K. pneumoniae* were selected for the amplification of the gene fragment covering the QRDR of the *gyrA* [26] and *parC* [27]. None of the *K. pneumoniae* isolates with a TMQR gene were susceptible to ciprofloxacin. The *gyrA* and *parC* amplicons were purified and subjected to bi-directional DNA sequencing by capillary electrophoresis (Macrogen, South Korea).

The chromatograms were visualized and processed in BioEdit software, and the sequences were then imported into MEGA11 software. In MEGA11 alignment explorer, the sequences were aligned by the ClustalW algorithm followed by codon-based nucleotide alignment. The substitutions in the QRDR of GyrA and ParC were determined by comparing the amino acid sequences of the isolates to the amino acid sequences of *E. coli* ATCC 25922 (GenBank Accession number NZ_CP032085 for *gyrA*, NZ_CP009072 for *parC*) and *Klebsiella pneumoniae* ATCC 13883 (GenBank Accession number DQ673325 for *gyrA*, KFJ75438 for *parC*).

### Data analysis

The data were collected in a Microsoft Excel spreadsheet and imported to IBM SPSS Statistics for Windows v.20 (IBM Corp, Armonk, NY). A chi-squared test of independence or Fisher exact test was performed to determine whether there was a significant relationship between TMQR and other categorical variables. The difference in ciprofloxacin MIC among TMQR positive and negative isolates was investigated by the Mann-Whitney U test. A cutoff value of ≤ 0.05 for the *P*-value was considered for statistical significance.

## Results

### Bacterial isolates

There were 522 unique uropathogens isolated from children with a UTI within the study period. Of the 522 isolates, there were 362 *E. coli* isolates and 74 *K. pneumoniae* isolates. Nalidixic acid resistance was present in 130/362 (35.9%) of *E. coli* and 24/74 (32.4%) of *K. pneumoniae*. Five *E. coli* and two *K. pneumoniae* isolates were not recovered in the sub-culture. The final sample size of this study was 147 isolates. The isolates included in this study were obtained from 100 female (68%) and 47 (32%) male children. The median (inter-quartile range (IQR)) age of the children was 6 (2–9) years.

### Antimicrobial susceptibility

The susceptibility results are in the supplementary material (Additional file 1: Fig. 1). For *E. coli*, 116/125 (92.8%) isolates were ciprofloxacin-resistant, 8/125 (6.4%) were intermediate, and only 1/125 (0.8%) isolate was susceptible. The median (IQR) ciprofloxacin MIC was 32 (16–128) µg/mL with values ranging from 0.25 to 512 µg/mL. The proportion of ciprofloxacin resistance, intermediate phenotype, and susceptibility among *K. pneumoniae* was 16/22 (72.7%), 5/22 (22.7%), and 1/22 (4.6%), respectively. The median (IQR) ciprofloxacin MIC for *K. pneumoniae* was 64 (0.75–128) µg/mL.

The proportion of susceptible *E. coli* was highest for nitrofurantoin (120/125, 96.0%) followed by imipenem (111/125, 88.8%) and piperacillin-tazobactam (96/125, 76.8%). *K. pneumoniae* displayed the highest susceptibility towards nitrofurantoin (n = 15/22, 68.2%), imipenem (13/22, 59.1%), piperacillin-tazobactam and amikacin (9/22, 40.9%) (Additional file 1: Fig. 1). Overall, 96/147 (65.3%) of the isolates were ESBL positive and 137/147 (93.2%) were MDR.

### Distribution of TMQR genes

Of 147 isolates, 74 were found to harbor TMQR genes (50.3%) (Tables 1 and 2). The prevalence of TMQR was slightly higher in males than females, 55.3% (26/47) vs. 48% (48/100), respectively. There were only 5 neonatal UTI cases caused by nalidixic acid-resistant organisms. Among the rest of the age groups, TMQR positivity was highest for adolescents (14/23, 60.9%) and lowest for children aged 6–12 years (24/58, 41.4%). The proportion of presence of TMQR was about 10% higher in *K. pneumoniae* (13/22, 59.1%) than in *E. coli* (61/125, 48.8%).

**Table 1:** Proportion of TMQR positivity stratified by sex, age groups, and pathogens

Variables	TMQR (%)	
	**Negative**	**Positive**
**Sex**		
Male	21 (44.7)	26 (55.3)
Female	52 (52.0)	48 (48.0)
**Age groups**		
Neonates (0–28 days)	1 (20.0)	4 (80.0)
Infants (1 month-2 years)	17 (44.7)	21 (55.3)
Preschool (3–5 years)	12 (52.2)	11 (47.8)
Children (6–12 years)	34 (58.6)	24 (41.4)
Adolescents (13–17 years)	9 (39.1)	14 (60.9)
**Organism**		
*Escherichia coli*	64 (52.2)	61 (48.8)
*Klebsiella pneumoniae*	9 (40.9)	13 (59.1)

The different TMQR gene combinations detected are in Table 2. A gel electrophoresis picture of the PCR amplification products of the representative TMQR positive isolates is shown in the supplementary material section (Additional file 2 Fig. 2) The most frequently detected TMQR gene was *aac(6’)-Ib-cr* present in 48 (32.7%) isolates. The *qnrB*, *qnrS*, *qnrA*, and *oqxAB* genes were detected in 23 (15.7%), 18 (12.3%), 1 (0.7%), and 1 (0.7%) isolate, respectively. Among 23 *qnrB* genes, 4 were *qnrB1* and the rest were *qnrB4*. All 18 *qnrS* and one *qnrA* gene were *qnrS1* and *qnrA1*, respectively. Of the 74 TMQR-positive isolates, 13 had two, and 2 had three TMQR genes. In *E. coli*, *aac(6’)-Ib-cr* (n = 39), *qnrB* (n = 16), and *qnrS* (n = 16) were among the most frequent, and similarly, for *K. pneumoniae* it was *aac(6’)-Ib-cr* (n = 9) and *qnrB* (n = 7).

**Table 2:** Co-occurrence of transferrable mechanisms of quinolone resistance determinants with β-lactamase gene combinations

TMQR-positive		
TMQR genes	ESBL or β-lactamase genes	Carbapenemase genes
*qnrB* (n = 9)		
*qnrB4* (n = 3)		
*qnrB1* (n = 1)	*bla*_CTX-M_	
*qnrB1* (n = 1)	*bla*_CTX-M_ *bla*_TEM_	
*qnrB4* (n = 2)	*bla*_CTX-M_ *bla*_TEM_	
*qnrB4* (n = 2)		*bla*_OXA-48_
*qnrS* (n = 15)		
*qnrS1* (n = 2)		
*qnrS1* (n = 3)	*bla*_CTX-M_	
*qnrS1* (n = 7)	*bla*_CTX-M_ *bla*_TEM_	
*qnrS1* (n = 1)	*bla*_CTX-M_	*bla*_OXA-48_
*qnrS1* (n = 2)		*bla*_OXA-48_
*aac(6’)-Ib-cr* (n = 35)		
*aac(6’)-Ib-cr* (n = 2)		
*aac(6’)-Ib-cr* (n = 10)	*bla*_CTX-M_	
*aac(6’)-Ib-cr* (n = 5)	*bla*_TEM_	
*aac(6’)-Ib-cr* (n = 12)	*bla*_CTX-M_ *bla*_TEM_	
*aac(6’)-Ib-cr* (n = 1)	*bla*_CTX-M_ *bla*_SHV_	
*aac(6’)-Ib-cr* (n = 1)	*bla*_TEM_	*bla*_NDM_ *bla*_OXA-48_
*aac(6’)-Ib-cr* (n = 3)		*bla*_NDM_
*aac(6’)-Ib-cr* (n = 1)		*bla*_NDM_ *bla*_OXA-48_
*qnrS1, qnrB4* (n = 1)		
*oqxAB, qnrS1* (n = 1)	*bla*_CTX-M_	
*aac(6’)-Ib-cr, qnrB* (n = 11)		
*aac(6’)-Ib-cr, qnrB1* (n = 1)	*bla*_CTX-M_	
*aac(6’)-Ib-cr, qnrB4* (n = 1)	*bla*_CTX-M_	
*aac(6’)-Ib-cr, qnrB4* (n = 5)	*bla*_CTX-M_ *bla*_TEM_	
*aac(6’)-Ib-cr, qnrB4* (n = 1)	*bla*_CTX-M_	*bla*_NDM_
*aac(6’)-Ib-cr, qnrB4* (n = 1)	*bla*_CTX-M_	*bla*_OXA-48_
*aac(6’)-Ib-cr, qnrB4* (n = 1)	*bla*_CTX-M_ *bla*_TEM_	*bla*_OXA-48_
*aac(6’)-Ib-cr, qnrB1* (n = 1)		*bla*_NDM_
*aac(6’)-Ib-cr, qnrB4, qnrS1* (n = 1)	*bla*_CTX-M_ *bla*_TEM_	
*aac(6’)-Ib-cr, qnrB4, qnrA1* (n = 1)	*bla*_CTX-M_	
**TMQR-negative**		
**Number (n)**	**ESBL or β-lactamase genes**	**Carbapenemase genes**
n = 36	*bla*_CTX-M_	
n = 1	*bla*_SHV_	
n = 1	*bla*_TEM_	
n = 1	*bla*_CTX-M_	*bla*_NDM_ *bla*_OXA-48_
n = 3		*bla*_OXA-48_
n = 2		*bla*_NDM_
n = 1		*bla*_NDM_ *bla*_OXA-48_

### GyrA and ParC substitutions

The amino acid substitution profiles observed in the QRDR of GyrA and ParC of *E. coli* and *K. pneumoniae* along with ciprofloxacin MIC values and the presence of TMQR genes are presented in Table 3. Twenty of 30 *E. coli* and three of five *K. pneumoniae* isolates had double residue substitutions (Ser-83→Leu and Asp-87→Asn) in GyrA and single substitution in ParC (Ser-80→Ile). Three *E. coli* isolates had double mutations in ParC (Ser-80→Ile and Glu-84→Val) in addition to GyrA double mutations (Ser-83→Leu and Asp-87→Asn). One *E. coli* isolate also had double-double mutations, but the alteration at the 84th position in ParC was from glutamic acid to glycine (Glu-84→Gly). One *K. pneumoniae* isolate had an alteration from aspartic acid to glycine at the 87th position in addition to Ser-83→Tyr in GyrA and Ser-80→Ile in ParC.

**Table 3:** Distribution of GyrA and ParC substitutions in 30 *E. coli* and 5 *K. pneumoniae* isolates

Organism	Isolate	Ciprofloxacin MIC (µl/mL)	TMQR	Amino acid residue substitutions in QRDR			
				GyrA		ParC	
				Ser-83	Asp-87	Ser-80	Glu-84
	EC147	0.25	*qnrS1*	-	-	-	-
	EC112	0.5	*qnrS1*	-	-	-	-
	EC49	0.5	*qnrS1*	-	-	-	-
	EC145	2	*qnrS1*	Leu	Asn	-	-
	EC114	2	*qnrS1*	Leu	-	-	-
	EC45	3	*qnrS1*	Leu	-	-	-
	EC35	16	*qnrS1*	Leu	Asn	Ile	-
	EC99	32	*aac(6’)-Ib-cr, qnrS1, qnrB4*	Leu	Asn	Ile	-
	EC142	32	*qnrS1*	Leu	Asn	Ile	-
	EC121	32	*aac(6’)-Ib-cr*	Leu	Asn	Ile	Val
	EC5	64	*qnrB4*	Leu	Asn	Ile	-
	EC66	64	*qnrB4*	Leu	Asn	Ile	-
*E. coli*	EC91	64	*aac(6’)-Ib-cr*	Leu	Asn	Ile	-
	EC64	64	*aac(6’)-Ib-cr*	Leu	Asn	Ile	-
	EC104	64	*qnrB4*	Leu	Asn	Ile	-
	EC43	64	*aac(6’)-Ib-cr*	Leu	Asn	Ile	-
	EC69	64	*acc(6’)-Ib-cr*	Leu	Asn	Ile	-
	EC76	128	*qnrB4*	Leu	Asn	Ile	-
	EC115	128	*aac(6’)-Ib-cr*	Leu	Asn	Ile	-
	EC139	128	*aac(6’)-Ib-cr, qnrB4*	Leu	Asn	Ile	-
	EC124	128	*qnrS1*	Leu	Asn	Ile	-
	EC108	128	*aac(6’)-Ib-cr*	Leu	Asn	Ile	Val
	EC134	128	*qnrB1*	Leu	Asn	Ile	-
	EC56	128	*aac(6’)-Ib-cr*	Leu	Asn	Ile	-
	EC67	256	*aac(6’)-Ib-cr*	Leu	Asn	Ile	-
	EC74	256	*aac(6’)-Ib-cr, qnrB4*	Leu	Asn	Ile	-
	EC46	256	*aac(6’)-Ib-cr, qnrB4*	Leu	Asn	Ile	Val
	EC12	256	*aac(6’)-Ib-cr*	Leu	Asn	Ile	Gly
	EC53	256	*aac(6’)-Ib-cr, qnrB4*	Leu	Asn	Ile	-
	EC125	512	*qnrB4*	Leu	Asn	Ile	-
	KP97	0.5	*qnrS1*	-	-	-	-
	KP146	64	*qnrB1*	Tyr	Gly	Ile	-
*K. pneumoniae*	KP41	128	*aac(6’)-Ib-cr, qnrB4*	Leu	Asn	Ile	-
	KP3	128	*aac(6’)-Ib-cr*	Leu	Asn	Ile	-
	KP40	256	*aac(6’)-Ib-cr, qnrB4*	Leu	Asn	Ile	-

Note: Ser = Serine, Leu = Leucine, Asp = Aspartic acid, Asn = Asparagine, Gly = Glycine, Ile = Isoleucine, Glu = Glutamic acid, Val = Valine, Tyr = Tyrosine. “-“ represents wild type (i.e. no substitution)

### Co-existence of TMQR with β-lactamase genes

The combinations of TMQR genes with β-lactamases are presented in Table 2. Among 74 TMQR-positive isolates, *bla*_CTX−M_, *bla*_TEM_, and *bla*_SHV_ were found in 51 (68.9%), 35 (47.3%), and 1 (1.4%) isolates, respectively. Four *K. pneumoniae* and twenty-five *E. coli* that harbored TMQR had both *bla*_CTX−M_ and *bla*_TEM_. A statistically significant association of TMQR positivity was observed with ESBL phenotype (p = 0.005) and the presence of *bla*_CTX−M_ (p = 0.037) and *bla*_TEM_ (p = 0.000) (Additional file 3: Table 1).

Of 23 imipenem non-susceptible isolates, carbapenemase production was observed in 21 isolates by mCIM. The proportion of *bla*_OXA−48_ and *bla*_NDM_ among TMQR positive and negative isolates were 12.2% (9 of 74) vs. 6.8% (5 of 73) and 9.5% (7 of 74) vs. 5.5% (4 of 73), respectively. This association was not statistically significant (Additional file 3: Table 1). No isolate was found to be positive for *bla*_KPC_, *bla*_IMP_, and *bla*_VIM_.

## Discussion

This study demonstrates alarmingly high levels of FQ resistance among *E. coli* and *K. pneumoniae* isolates causing UTI in children attending the outpatient department of Siddhi Memorial Hospital, Bhaktapur, Nepal. Half of the isolates were TMQR positive which suggests that TMQR genes may have an important role in the emergence of quinolone resistance in *E. coli* and *K. pneumoniae* isolates within our study population. TMQR genes were found to have a statistically significant association with two β-lactamases, *bla*_CTX−M_ and *bla*_TEM_.

We found a high prevalence of TMQR in diverse gene combinations among study isolates. Similar high proportions of the TMQR genes have been reported in previous studies, while few studies have comparatively lower proportions. The proportion of TMQR positivity among FQ-resistant isolates we report, 68/132 (51.5%) of ciprofloxacin-resistant isolates, is similar to a study from the Netherlands (29/ 56, 51.8%) [28], higher than in Korea (13/122, 10.7%) [29], Taiwan (37/248, 14.9%) [30], and China (137/302, 45.4%) [31], and lower than in Iran (54/60, 90%) [32], South Africa (47/48, 98%) [33], and Egypt (90/90, 100%) [34]. Studies from China, Korea, and Taiwan investigated solely the *E. coli* isolates and the Iran study included *E. coli* and *K. pneumoniae*. The rest of the three studies had various *Enterobacterales* isolates. The proportion and distribution of TMQR genes vary among different studies possibly due to the heterogeneity in the isolate selection criteria, the specific TMQR genes investigated, and the study population. Also, the actual proportion of TMQR could be slightly higher than reported in this study among uropathogens at our institution because they can be present even among nalidixic acid-susceptible *Enterobacterales* [11]. Since we only included nalidixic-resistant isolates, we might have missed isolates with such phenotype.

The distribution of the TMQR genes observed in this study is consistent with the general distribution reported by previous studies. We found the highest prevalence for three TMQR genes; *aac(6’)-Ib-cr* (n = 48, 32.7%), *qnrB* (n = 23, 15.7%), and *qnrS* (n = 18, 12.3%) (Table 2). In line with this study, the *aac(6’)-Ib-cr* gene was the most common TMQR gene among FQ-resistant *E. coli* in the investigation in South Korea (11/122, 9%) [29], China (74/302, 24.5%) [31], and Netherlands (23/56, 41.1%) [28]. Studies from Iran, South Africa, China, Taiwan, and Egypt found *qnrB* and *qnrS* as the most common compared to other *qnr* genes investigated among FQ-resistant clinical isolates, in agreement with our findings [30–34]. On the other hand, the prevalence of *oqxAB* or *oqxA/B* in Iran [*oqxA*: 22/60 (36.7%), *oqxB*: 31/60 (51.7%)], South Africa [*oqxA*: 20/48 (41.7%), *oqxB*: 43/48 (89.6%)], China [*oqxAB*: 19/302 (6.3%)], and Taiwan [*oqxAB*: 15/248 (6.1%)] is contrary to our findings; we only found one *E. coli* isolate with *oqxAB* gene among 125 isolates investigated [30–33]. No isolate was found to harbor *qepA* gene similar to the study in the Netherlands and Taiwan [28, 30], but 3/60 (5.0%), 9/90 (10.0%), and 36/302 (11.9%) of FQ-resistant isolates were found to possess *qepA* in Iran, Egypt, and China, respectively [31, 32, 34]. Overall, our data in conjunction with the previous findings suggest that *aac(6’)-Ib-cr* and the two *qnr* genes, *qnrB* and *qnrS*, are the most prevalent TMQR genes among Q/FQ-resistant *Enterobacterales* in general. A recent study demonstrated that possession of *aac(6’)-Ib-cr* gives a selective advantage to *E. coli* ST131 in the presence of ciprofloxacin [35]. In addition, alone or in combination with chromosomal mutations, QnrS1 has been shown to increase bacterial fitness while QnrA1 and QepA2 decrease fitness [11]. These observations could explain the predominance of *aac(6’)-Ib-cr* and *qnrS*, and the low prevalence of *qnrA* and *qepA*.

Our data show that TMQR-positive isolates have higher FQ MIC than those that lack them similar to studies from Iran and Korea [36, 37]. The ciprofloxacin MIC values were significantly higher in TMQR positive isolates (Median = 64 µg/mL, n = 74) compared to TMQR negative isolates (Median = 32 µg/mL, n = 73) (Mann-Whitney U = 1969, Z=-2.871, p = 0.004, but with a small effect size of r = 0.24). Results from the analysis of 35 representative isolates suggest that the concomitant presence of GyrA and ParC substitutions accompanies TMQR genes to result in high levels of FQ resistance (Table 3). Similar findings of multiple substitutions in GyrA and ParC along with TMQR genes leading to high FQ resistance have been shown in other studies [34, 38].

The statistically significant association of TMQR with ESBL observed in this study mirrors previous findings from several other studies [36, 37, 39, 40]. The β-lactamase genotypes, *bla*_TEM_ and *bla*_CTX−M_, showed an independent association with TMQR, but the difference in the proportion of *bla*_TEM_ was remarkably high between TMQR positive and TMQR negative group (47.3% vs. 1.4%) (Additional file 3: Table 1). Notably, of 57 ESBL-producing TMQR positive isolates, 29 (50.9%) co-harbored both *bla*_TEM_ and *bla*_CTX−M_ while no TMQR negative isolate had more than one β-lactamase gene (Table 2). These observations suggest that the association of β-lactamase and TMQR is driven by the co-existence of multiple β-lactamase genes rather than a single genotype in the study population. In a study from Iran investigating UTI caused by *Enterobacterales*, 72 (43.6%) isolates had the co-existence of *bla*_CTX−M_ and *bla*_TEM_ among 165 ESBL-producing TMQR positive *E. coli* and *K. pneumoniae* isolates [36]. In contrast, among 155 ESBL-positive TMQR harboring *K. pneumoniae* (originating from various clinical specimens) in a study in Algeria, all 155 isolates had *bla*_CTX−M_ only [41]. Geographic, demographic, and differences in clinical specimens could account for this disparity. The predominance of *bla*_CTX−M_ as the most common ESBL gene associated with TMQR is in an agreement with both Iranian and Algerian studies. We also demonstrate carbapenemase genes among TMQR-positive isolates, in contrast to previous findings; most studies either had no or negligible TMQR-positive isolate resistant to carbapenem [31, 34, 39, 42]. Two-thirds (14/21, 66.7%) of the carbapenemase-producing isolates were TMQR-positive. Although, this was not a statistically significant association (Additional file 3: Table 1).

WHO’s GLASS report 2022 showed that more than 90% of antimicrobial use in Nepal in 2018 was attributed to oral administration reflecting their use in the community setting [43]. Ciprofloxacin and cefixime were the second and third most consumed oral antimicrobials, respectively. Pathogens harboring resistance mechanisms for either or both of these two antimicrobials most likely thrived under such high selective pressure in the community. A recent study from a tertiary care center in Nepal with a large sample size (n = 2153) showed a high prevalence of isolates with overlapping resistance to extended-spectrum cephalosporin and fluoroquinolone in both inpatient and outpatient settings that is consistent with the hypothesis that these two groups of genes are co-spreading in Nepal [44]. TMQR and ESBL genes can be located in the same conjugative plasmid with other antimicrobial-resistant determinants, and this facilitates their simultaneous spread and contributes to the emergence of MDR [11]. Such co-localization implies that the use of either quinolones or β-lactams could also promote the selection of these strains as suggested in a study from Vietnam [45]. Considering that fluoroquinolone is typically avoided in children, high levels of fluoroquinolone resistance may be explained by the high prevalence of such strains, promoted by selective pressure in the community, and by the spread of strains with co-localization of TMQR and β-lactamases within the same conjugative plasmids.

TMQR genes seem to have community origin [42, 46], and several studies have shown that commensal gut microbiota frequently harbors these genes [46, 47], as do isolates from other body surfaces [48]. With the growing appreciation of the involvement of the gut microbiome [49] and urinary microbiome in causing UTI [50], the detection and characterization of TMQR genes from the microbiome of these niches could be a future investigation at our institution. Characterization of the genetic background of TMQR (such as TMQR copy number, and expression level), conjugation experiments, phylogrouping, and MLST could be another aspect of focus for further research. The lack of data on whether the patients had consumed antimicrobials prior to hospital visits is a limitation of this study. In addition, we have not characterized other mechanisms of quinolone resistance, such as chromosomal efflux pumps, permeability alterations, and the role of biofilms.

## Conclusions

Our study demonstrates alarmingly high levels of FQ resistance among *E. coli* and *K. pneumoniae* causing UTI in Nepalese children indicating the presence of high selective pressure in the community to promote their resistance. High prevalence and diversity in combinations of TMQR genes among quinolone-resistant *E. coli* and *K. pneumoniae* suggest an important role of these genes in the emergence of Q/FQ resistance. Also, the findings of this study highlight the dissemination of TMQR along with β-lactamases among the pediatric population in Nepal, amplifying multidrug resistance. The increase in FQ resistance is a challenge for the management of UTIs. Comprehensive prospective surveillance of antimicrobial resistance in these common pathogens will be necessary to understand the origin and spread of TMQR genes and to devise strategies to mitigate the emergence of further resistance.

### Electronic supplementary material

Below is the link to the electronic supplementary material.

Supplementary Material 1

Supplementary Material 2

Supplementary Material 3

## Data Availability

The datasets generated and/or analysed during the current study are available in the GenBank repository (https://www.ncbi.nlm.nih.gov/genbank/), accession numbers OR271074 to OR271143.

## List of abbreviations

UTI: Urinary tract infection
DNA: Deoxyribonucleic acid
QRDR: Quinolone resistance-determining region
ICU: Intensive care unit
CFU: Colony forming unit
TMQR: Transferrable mechanisms of quinolone resistance
WBC: White blood cells
HPF: High-power field
CLSI: Clinical Laboratory Standards Institute
ESBL: Extended-spectrum β-lactamase
