# Supplementary material for: Characterization of Transferrable Mechanisms of Quinolone Resistance (TMQR) among Quinolone-resistant Escherichia coli and Klebsiella pneumoniae causing Urinary Tract Infection in Nepalese Children
Source: BMC Pediatr. 2023 Sep 13;23:458. doi: 10.1186/s12887-023-04279-5 (PMC10498618; doi:10.1186/s12887-023-04279-5)
Supplement: Supplementary file 1 — Supplementary Material 1 [file 12887_2023_4279_MOESM1_ESM.docx]

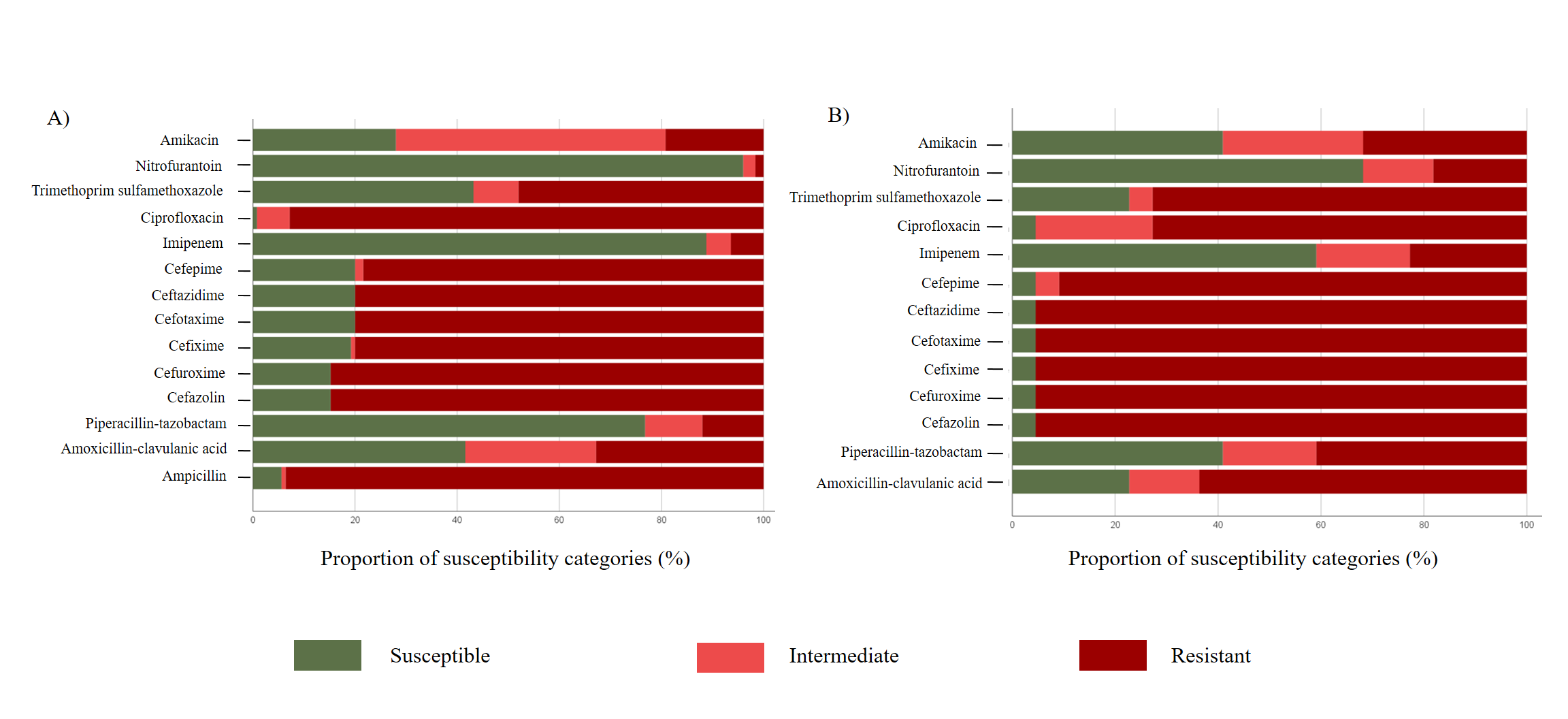


**Additional file 1 Fig 1. Stacked bar chart illustrating the results of antimicrobial susceptibility testing against various antimicrobials tested by disk diffusion method for urinary isolates of A) *Escherichia coli* B) *Klebsiella pneumoniae***
