# Supplementary material for: Characterization of Transferrable Mechanisms of Quinolone Resistance (TMQR) among Quinolone-resistant Escherichia coli and Klebsiella pneumoniae causing Urinary Tract Infection in Nepalese Children
Source: BMC Pediatr. 2023 Sep 13;23:458. doi: 10.1186/s12887-023-04279-5 (PMC10498618; doi:10.1186/s12887-023-04279-5)
Supplement: Supplementary file 2 — Supplementary Material 2 [file 12887_2023_4279_MOESM2_ESM.docx]

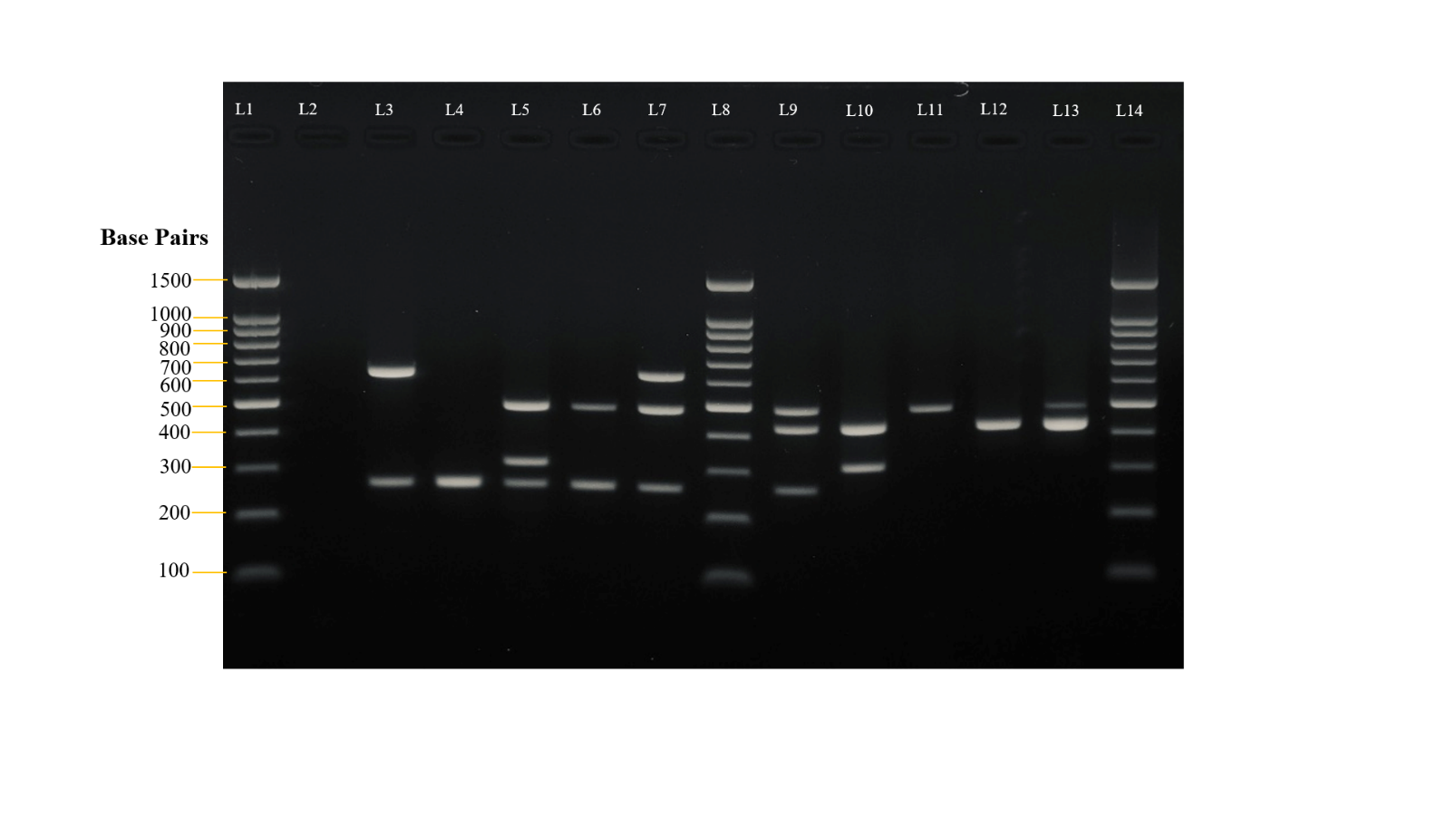


**Additional file 2 Fig 2. Gel electrophoresis picture showing PCR amplification products of TMQR genes detected in the study**. Note: L1, L8 and L14: 100bp molecular marker, L2: No template control, L3: Positive control (*qnrA and aac(6’)-Ib-cr*), L4: *aac(6’)-Ib-cr* (260bp), L5: *qnrB* (488bp), *oqxAB* (313bp), *aac(6’)-Ib-cr* (260bp), L6: *qnrB* (488bp) and *aac(6’)-Ib-cr* (260bp), L7: *qnrA* (630bp), *qnrB* (488bp), and *aac(6’)-Ib-cr* (260bp), L9: *qnrB* (488bp), *qnrS* (428bp), and *aac(6’)-Ib-cr* (260bp), L10: *qnrS* (428bp) and *oqxAB* (313bp), L11: *qnrA* (630), L12: *qnrS* (428bp), L13: *qnrB* (488bp) and *qnrS* (428bp)
