# Supplementary material for: Characterization of Transferrable Mechanisms of Quinolone Resistance (TMQR) among Quinolone-resistant Escherichia coli and Klebsiella pneumoniae causing Urinary Tract Infection in Nepalese Children
Source: BMC Pediatr. 2023 Sep 13;23:458. doi: 10.1186/s12887-023-04279-5 (PMC10498618; doi:10.1186/s12887-023-04279-5)
Supplement: Supplementary file 3 — Supplementary Material 3 [file 12887_2023_4279_MOESM3_ESM.docx]

**Additional file 3 Table 1:**

**Association of TMQR with ESBL phenotype and β-lactamases assessed by chi-square test of independence or Fisher exact test**

| **Characteristics** | **TMQR, n (%)** | | **P-value^*^** |
| --- | --- | --- | --- |
|  | **Positive (n=74)** | **Negative (n=73)** |  |
| ESBL phenotype | 57 (77.0) | 39 (53.4) | 0.005 |
| ESBL genotype |  |  |  |
| *bla*_CTX-M_ | 51 (68.9) | 37 (50.7) | 0.037 |
| *bla*_TEM_ | 35 (47.3) | 1 (1.4) | 0.000 |
| *bla*_SHV_ | 1 (1.4) | 1 (1.4) | 1.000^A^ |
| Carbapenemase |  |  |  |
| *bla*_OXA-48_ | 9 (12.2) | 5 (6.8) | 0.414 |
| *bla*_NDM_ | 7 (9.5) | 4 (5.5) | 0.546 |

^*^ *P*-value calculated by Chi-squared test unless specified

^A^ *P*-value calculated by Fisher exact test
